# Characterization of Clinical *Escherichia coli* Strains Producing a Novel Shiga Toxin 2 Subtype in Sweden and Denmark

**DOI:** 10.3390/microorganisms9112374

**Published:** 2021-11-17

**Authors:** Xiangning Bai, Flemming Scheutz, Henrik Mellström Dahlgren, Ingela Hedenström, Cecilia Jernberg

**Affiliations:** 1 Department of Laboratory Medicine, Division of Clinical Microbiology, Karolinska Institutet, 141 52 Stockholm, Sweden; xiangning.bai@ki.se; 2 Division of Laboratory Medicine, Oslo University Hospital, 0372 Oslo, Norway; 3 The International *Escherichia* and *Klebsiella* Centre, Statens Serum Institut, 2300 Copenhagen, Denmark; FSC@ssi.dk; 4 County Council Department of Communicable Disease Control and Prevention, Region Västra Götaland, 411 18 Gothenburg, Sweden; henrik.mellstrom@vgregion.se; 5 Public Health Agency of Sweden, 171 82 Solna, Sweden; ingela.hedenstroms@gmail.com

**Keywords:** Shiga Toxin-Producing *Escherichia coli*, Shiga toxin subtype, communicable disease, diarrhea, surveillance

## Abstract

Shiga toxin (Stx) is the key virulence factor in the Shiga Toxin-Producing *Escherichia coli* (STEC), which can cause diarrhea and hemorrhagic colitis with potential life-threatening complications. There are two major types of Stx: Stx1 and Stx2. Several Stx1/Stx2 subtypes have been identified in *E. coli*, varying in sequences, toxicity and host specificity. Here, we report a novel Stx2 subtype (designated Stx2m) from three clinical *E. coli* strains isolated from diarrheal patients and asymptomatic carriers in Sweden and Denmark. The Stx2m toxin was functional and exhibited cytotoxicity in vitro. The two Swedish Stx2m-producing strains belonged to the same serotype O148:H39 and Multilocus Sequencing Typing (MLST) Sequence Type (ST) 5825, while the Danish strain belonged to the O96:H19 serotype and ST99 type. Whole-genome sequencing (WGS) data analysis revealed that the three Stx2m-producing strains harbored additional virulence genes and the macrolide resistance gene *mdf* (A). Our findings expand the pool of Stx2 subtypes and highlight the clinical significance of emerging STEC variants. Given the clinical relevance of the Stx2m-producing strains, we propose to include Stx2m in epidemiological surveillance of STEC infections and clinical diagnosis.

## 1. Introduction

Shiga Toxin-Producing *Escherichia coli* (STEC) can cause various gastrointestinal diseases in humans, ranging from asymptomatic carriers to life-threatening Hemolytic Uremic Syndrome (HUS) [1]. The major virulence factors of STEC are Shiga toxins 1 and 2 (Stx1 and Stx2, corresponding genes are referred to as *stx1* and *stx2*), which target human podocytes (visceral epithelial cells) and renal microvascular endothelial cells, where they inhibit protein synthesis [2]. Stx1/Stx2 can further be divided into a number of subtypes, which differ in receptor binding, toxin potency and host specificity. Stx2 is more heterogeneous and virulent than Stx1, with Stx2a (with or without Stx2c) being considered more pathogenic to humans than other Stx subtypes [3]. However, some rare human Stx2 subtypes, such as Stx2e and Stx2f, have also been related to severe clinical symptoms such as HUS [4,5]. Since the standardized Stx nomenclature [6] Stx2a to Stx2g was established, several new Stx2 subtypes have been identified: Stx2h (Acc. No. CP022279) [7], Stx2i (Acc. No. FN252457) [8], Stx2j (Acc. No. MZ571121) (Stanton, E., Cebelinski, E. and Wang, X., Direct submission), Stx2k (Acc. No. CP041435) [9] and Stx2l (Acc. No. AM904726) [10].

In our present study, we describe the identification of a novel Stx2 subtype from clinical STEC strains isolated from diarrheal patients and an asymptomatic carrier in Sweden and Denmark. We performed whole-genome sequencing (WGS) to characterize the genomic features of the new Stx2 subtype and assess the Shiga toxin-production capability of the isolates.

## 2. Materials and Methods

### 2.1. Ethics Statement

Ethical approval was not required as the investigation was performed under the mandate of the Public Health Agency of Sweden and the Statens Serum Institut (SSI) in Denmark in their respective remits for national communicable disease surveillance and control in the interest of public health.

### 2.2. Collection of STEC Strains and Clinical Data

In Sweden and Denmark, STEC isolates are submitted from the clinical microbiological laboratories to the Public Health Agency of Sweden and to the SSI, respectively, for confirmation and further typing as part of the national microbial surveillance programs. The two Swedish cases were a male (aged 70) and a female (aged 69). One case was reported to have mild gastrointestinal symptoms and a stool sample was routinely taken because the patient had been abroad. The other case was identified during source tracing and was asymptomatic. Their infections were reported as having been contracted in Namibia. The Danish case was a male (aged 88), who was hospitalized on suspicion of a urinary tract infection (UTI). The STEC was isolated from a stool specimen and the preliminary diagnosis indicated *stx2d*, which is considered as a HUS-associated Stx subtype.

### 2.3. Whole-Genome Sequencing (WGS) and Genome Assembly

DNA extracted from the two Swedish STEC strains was analyzed using the Ion Torrent S5 XL platform (Thermo Fisher Scientific, Waltham, Massachusetts, USA) as previously described [11]. The sequencing reads were de novo assembled with Spades (v3.13.1) [12] in “careful mode” to correct mismatches. The Danish strain was sequenced using an Illumina Nextseq (Illumina, San Diego, CA, USA) as previously described [13]. The sequencing reads were de novo assembled with SKESA (version 2.3.0) with default settings [14].

### 2.4. WGS-Based Molecular Characterization

The WGS assemblies were analyzed using the SerotypeFinder, VirulenceFinder and ResFinder databases (https://cge.cbs.dtu.dk/services/ (accessed on 1 September 2021)) for the determination of serotypes, virulence genes and antimicrobial resistance genes, respectively. Multilocus sequence typing (MLST) was conducted in silico using the online tool provided by the Warwick *E. coli* MLST scheme website (https://enterobase.warwick.ac.uk/species/ecoli/allele_st_search (accessed on 1 September 2021)).

### 2.5. Stx Subtyping

The Stx subtypes of STEC isolates were initially determined by ABRicate version 0.8.10 (https://github.com/tseemann/abricate (accessed on 1 March 2020) with the default parameters. Briefly, an in-house *stx* subtyping database was created with the ABRicate by including representative nucleotide sequences of all identified Stx1 and Stx2 subtypes. The assemblies were then searched against the *stx* subtyping database. For the *stx* genes that yield an identity below 96% with the nearest known *stx* subtype, the full nucleotide sequences were extracted and compared to the GenBank database with the NCBI Blast tool. The representative nucleotide sequences of all the Stx2 subtypes and variants (*stx2a*-*stx2l*) described previously were downloaded from the GenBank. The amino acid sequences for the combined A and B subunits of Stx2 holotoxin were translated from the open reading frames. The full nucleotide and amino acid sequences were aligned to calculate the genetic distances between *stx2*/Stx2 sequences. Evolutionary unrooted trees were created from maximum parsimony cluster analysis using 100 bootstrap simulations. In addition, the amino acid sequences were analyzed for sequence motifs that support the phylogenetic analyses using BioNumerics version 7.6 (Applied Maths, Ghent, Belgium), as previously described [6].

### 2.6. Detection of Shiga Toxin Production

Cytotoxic activity on Vero cells is a common characteristic of STEC strains due mainly to the production of Shiga toxins [15]. Stx2 production was thus determined by the Vero cell cytotoxicity assay (VCA) as previously described [16]. The experiment was performed in triplicate and then three times independently for each strain.

### 2.7. Antimicrobial Susceptibility Testing

Antimicrobial susceptibility testing was performed as a minimum inhibitory concentration (MIC) determination using the Sensititre broth microdilution system (Surveillance *Salmonella*/*E. coli* EUVSEC3 AST Plate; Trek Thermo-Fisher). Inoculation and incubation procedures were in accordance with the CLSI guidelines [Clinical and Laboratory Standards Institute, Wayne, PA, USA] and the European standard ISO 20776-1:2006.

### 2.8. Data Availability

The raw sequencing data of the three Stx2m-producing strains are available at The European Nucleotide Archive (ENA) under the accession numbers shown in Table 1.

## 3. Results

### 3.1. Identification of a Novel Stx2 Subtype in Clinical STEC Strains

**Figure 1:**
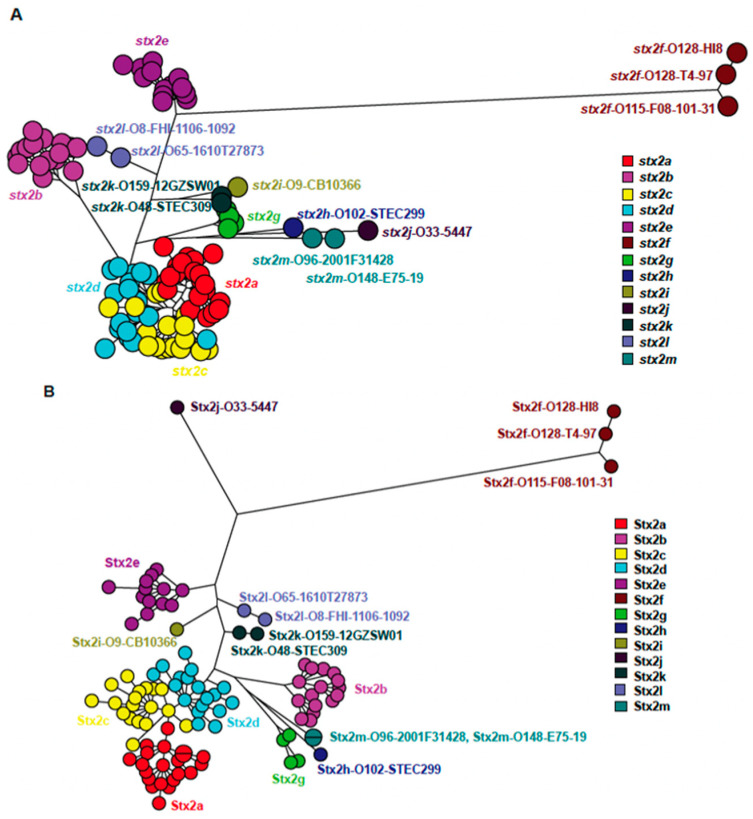
Comparison of nucleotide (**A**) and amino acid (**B**) sequences of different Stx2 subtypes using maximum parsimony cluster analysis. Stx2a, Stx2b, Stx2c, Stx2d, Stx2e, Stx2f, Stx2g adapted from Scheutz et al., 2012. Stx2h (Bai et al., 2018), Stx2i (FN252457), Stx2j (MZ571121), Stx2k (Yang et al., 2020) and Stx2l (EFSA BIOHAZ Panel, 2020).

The in-house *stx*-subtyping based on whole-genome sequences showed that the *stx2* sequences of the three clinical isolates shared less than 93% nucleic acid sequence identities with other Stx2 subtypes. The complete nucleic acid sequence of *stx2* gene of the three isolates was extracted from genome assemblies and compared against the Genbank database using NCBI BLAST, showing the highest similarity of 93.35% with *Escherichia coli* strain 646 chromosome (CP023200.1). The two Swedish isolates shared an identical *stx2* nucleic acid sequence while the Danish isolate differed by one synonymous nucleotide at position 486 (A->G). When comparing sequences of Stx2 holotoxin, Stx2m shared 63.4 to 92.6% similarity with the other 12 described Stx2 subtypes at nucleic acid level and 72.4 to 93.8% at amino acid level (Table 2). Phylogenetic trees (Figure 1. see Supplemental Material Figure S1 for extended version of this tree) demonstrated that the two unique Stx2 sequences from the three STEC isolates form a distinct lineage from the described Stx2 subtypes and variants (Stx2a to Stx2l). These results suggest that the three STEC strains harbor a novel Stx2 subtype. Based on the new nomenclature for Stx2 [6], the new Stx2 subtype was designated Stx2m.

As part of contact tracing, the three Stx2m-STEC strains were isolated from two patients with diarrhea and one asymptomatic carrier (Table 1). 

All three strains were positive in the Vero cell assay (VCA).

### 3.2. Genetic Features of Stx2m-Producing Strains

The genome size of the two Swedish Stx2m-producing isolates was 5,514,783 bp and 5,565,491 bp, respectively, with an average genomic GC content of 50.39 and 50.43, respectively. The Danish isolate had a genome size of 4,948,862 bp with a GC content of 50.94 (Table 1). The two Swedish isolates were assigned to the same serotype O148:H39 and MLST type ST5825, while the Danish isolate belonged to serotype O96:H19 and MLST type ST99 (Table 1).

WGS analysis showed that the two Swedish isolates harbored the same virulence genes: *stx2*, *iha*, *astA*, *terC*, *hra*, *chuA*, *eilA, traT, kpsMII_K5, mchB*, *mchC* and *mchF*. The Danish isolate was different and tested positive for *stx2*, *astA*, *celb*, *iss*, *lpfA*, *ompT* and *terC* (Table 3). The three Stx2m-STEC isolates tested positive for the macrolide resistance gene *mdf* (A) but were susceptible to all antibiotics, azithromycin included.

## 4. Discussion

Shiga toxin (Stx) is the main characteristic defining STEC and is the key virulence factor in STEC that causes HUS. Stx subtyping is not only useful for STEC characterization but is also valuable for diagnosis and risk assessment. Various Stx subtypes can exhibit significant differences in pathogenic potential and, thereby, clinical outcome. It is well known that Stx2a is frequently associated with a higher risk of HUS development, while Stx2e, Stx2f and Stx2g are present mainly in strains isolated from patients with uncomplicated diarrhea or non-human sources. In our present study, three Stx2m-producing STEC strains were isolated from patients in clinical settings. The two Swedish Stx2m-STEC isolates harbored the same set of virulence genes. Besides *stx2*, genes encoding adherence (*iha)*, heat-stable enterotoxin 1 (*astA*), invasion protein (*eilA*), etc. were present. The Danish isolate differed by carrying the adherence gene *lpfA* and genes encoding heat-stable enterotoxin 1 (*astA*), increased serum survival (*iss*), tellurium ion resistance protein (*terC*), etc. These results indicate that the clinical significance of the Stx2m-producing strains should be noted. When specific criteria are met, protein synthesis inhibitors such as azithromycin followed by cell-wall synthesis inhibitors have been suggested as an antibiotic treatment for STEC-infected patients [17]. All three Stx2m-STEC strains from this study were susceptible to azithromycin. In addition, the new Stx2m subtype was identified in two different serotypes, showing the mobility and spread of the phages carrying the genes.

The different gene variants of the Shiga toxin subtypes are defined by their sequence relatedness within Stx1 and Stx2, respectively. A new Stx subtype is defined when differences in the sequence lead to a change in one or more amino acids. It should be noted that the aa motifs in the Stx2m sequence are identical to those in the Stx2d in the C-terminal part of the A subunit, which has been associated with the activatable characteristic (SLYTTGE at positions 313–319) but not with the “END” motif (positions 16–18) in the mature B subunit. Therefore, we do not expect the Stx2m toxin to be activated by elastase.

In conclusion, the novel Stx2m subtype should be included in molecular typing of STEC strains and in epidemiological surveillance of STEC infections.

## Figures and Tables

**Table 1:** Characterization of three Stx2m-producing STEC isolates.

	E75_19	E79_19	2001F31428
**Clinical information**			
Source	Diarrheal patient	Healthy contact	Diarrheal patient
Infected region	Sweden	Sweden	Denmark
Isolation year	2019	2019	2020
**Genetic characteristics**			
Serotype	O148:H39	O148:H39	O96:H19
Stx2 subtype	Stx2m	Stx2m	Stx2m
Sequence type	5825	5825	99
Genome size (bp)	5,514,783	5,565,491	4,948,862
CG %	50.39	50.43	50.94
Accession number	SAMEA7019263	SAMEA7019264	SAMEA6873236

**Table 2:** Nucleotide\amino acids identities (%) between Stx2m and representatives of other described Stx2 subtypes.

Nucleotide\ Amino Acids	1	2	3	4	5	6	7	8	9	10	11	12	13
1. Stx2a		92.6	98.8	97.5	92.6	72.4	95.6	92.4	93.6	90.1	95.6	95.1	**93.3**
2. Stx2b	91.3		92.9	93.6	90.6	70.9	92.1	92.6	89.9	89.6	92.6	90.6	**92.1**
3. Stx2c	98.4	91.6		97.8	92.1	71.7	94.9	92.1	92.9	90.3	95.8	94.4	**92.6**
4. Stx2d	96.8	92.9	97.4		93.1	72.4	95.6	92.9	93.9	91.1	97.1	95.3	**92.9**
5. Stx2e	91.5	88.5	91.1	91.3		75.6	93.6	91.1	95.8	88.6	95.6	95.8	**90.9**
6. Stx2f	61.7	62.6	61.2	61.2	69.1		72.9	71.9	72.9	73.2	72.7	72.9	**72.4**
7. Stx2g	93.9	90.7	92.7	93.8	91.7	63.5		92.9	94.6	88.8	95.1	94.1	**93.8**
8. Stx2h	91.2	91.8	91.1	91.7	89.5	63.6	91.4		92.6	90.1	93.3	91.6	**91.9**
9. Stx2i	92.9	88.3	91.9	92.5	94.4	63.5	94.3	91.5		88.3	96.8	95.8	**91.4**
10. Stx2j	88.3	88.2	88.4	88.8	87.2	63.0	87.2	91.6	87.6		90.8	90.6	**87.8**
11. Stx2k	94.2	90.5	94.5	96.0	93.2	62.0	92.5	92.4	96.4	89.3		96.6	**92.1**
12. Stx2l	95.3	89.1	94.6	94.6	94.3	62.0	92.4	90.5	94.9	88.2	95.3		**90.1**
13. **Stx2m**	**92.6**	**89.8**	**91.5**	**90.7**	**87.9**	**63.4**	**91.0**	**91.5**	**89.7**	**88.2**	**90.0**	**89.0**	

1. EDL933 (X07865), 2. EH250 (AF043627), 3. 031 (L11079), 4. C165-02 (DQ059012), 5. S1191 (M21534), 6. F08-101-31 (AB472687), 7. 7v (AY286000), 8. STEC299 (CP022279), 9. CB10366 (FN252457), 10. 5447 (MZ571121), 11. STEC309 (CP041435), 12. FHI 1106-1092 (AM904726) and 13. E75_19 (SAMEA7019263). Bold values highlight the sequence identities of newly identified Stx2m subtype with other Stx2 subtypes.

**Table 3:** Presence of virulence genes in three Stx2m-producing STEC strains.

Genus	Function	E75_19	E79_19	2001F31428
*stx2*	Shiga toxin 2	+	+	+
*iha*	Adherence protein	+	+	-
*astA*	Heat-stable enterotoxin 1	+	+	+
*lpfA*	Long polar fimbriae	-	-	+
*iss*	Increased serum survival	-	-	+
*celb*	Endonuclease colicin E2	-	-	+
*ompT*	Outer membrane protease	-	-	+
*terC*	Tellurium ion resistance protein	+	+	+
*hra*	Heat-resistant agglutinin	+	+	-
*chuA*	Outer membrane hemin receptor	+	+	-
*eilA*	HilA (an invasion protein) homolog	+	+	-
*traT*	Outer membrane protein complement resistance	+	+	-
*kpsMII_K5*	Polysialic acid transport protein	+	+	-
*mchB*	Microcin H47 part of colicin H	+	+	-
*mchC*	Microcin H47 biosynthesis protein	+	+	-
*mchF*	ABC transporter protein MchF	+	+	-

## Data Availability

The raw sequencing data of the three Stx2m-producing strains is available at the European Nucleotide Archive (ENA) under the accession numbers shown in Table 1.

## Supplementary Materials

The following are available online at https://www.mdpi.com/article/10.3390/microorganisms9112374/s1, Figure S1. Five main clusters of Stx2a-Stx2g and representative sequences of six newly designated Stx2h-Stx2m subtypes: Neighbour-Joining cluster analysis of 102 unique sequences as described in the text and the proposed new designations.
