# Supplementary material for: Characterization of Clinical Escherichia coli Strains Producing a Novel Shiga Toxin 2 Subtype in Sweden and Denmark
Source: Microorganisms. 2021 Nov 17;9(11):2374. doi: 10.3390/microorganisms9112374 (PMC8625421; doi:10.3390/microorganisms9112374)
Supplement: Supplementary file 1 [file microorganisms-09-02374-s001.zip › microorganisms-1430817-supplementary.pdf]

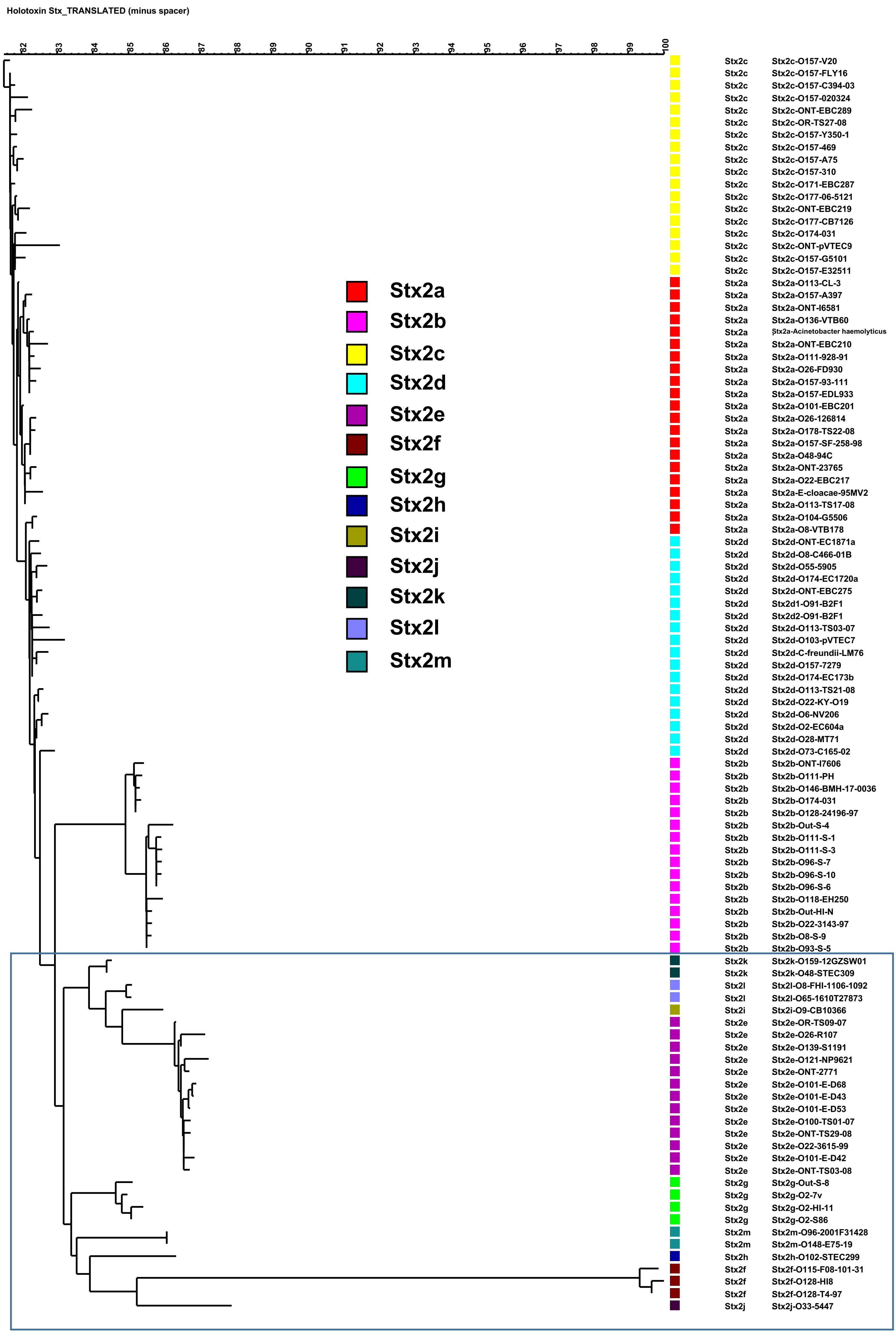

Figure S1. Five main clusters of Stx2a-Stx2g and representative sequences of six newly designated Stx2h-Stx2m subtypes: Neighbour-Joining cluster analysis of 102 unique sequences as described in the text and the proposed new designations.
